# Geographic variation in projected US forest aboveground carbon responses to climate change and atmospheric deposition

**DOI:** 10.1088/1748-9326/ad2739

**Published:** 2024-02-20

**Authors:** Aspen Reese, Christopher M Clark, Jennifer Phelan, John Buckley, James Cajka, Robert D Sabo, George Van Houtven

**Affiliations:** 1 American Association for the Advancement of Science (AAAS) Science and Technology Policy Fellow, at the US Environmental Protection Agency, Office of Research and Development, Center for Public Health and Environmental Assessment, Washington, DC, United States of America; 2 US Environmental Protection Agency, Office of Research and Development, Center for Public Health and Environmental Assessment, Washington, DC, United States of America; 3 RTI International, Research Triangle Park, NC, United States of America

**Keywords:** aboveground carbon, atmospheric deposition, climate change, forests

## Abstract

Forest composition and ecosystem services are sensitive to anthropogenic pressures like climate change and atmospheric deposition of nitrogen (N) and sulfur (S). Here we extend recent forest projections for the current cohort of trees in the contiguous US, characterizing potential changes in aboveground tree carbon at the county level in response to varying mean annual temperature, precipitation, and N and S deposition. We found that relative to a scenario with N and S deposition reduction and no climate change, greater climate change led generally to decreasing aboveground carbon (mean −7.5% under RCP4.5, −16% under RCP8.5). Keeping climate constant, reduced N deposition tended to lessen aboveground carbon (mean −7%), whereas reduced S deposition tended to increase aboveground carbon (+3%) by 2100. Through mid-century (2050), deposition was more important for predicting carbon responses except under the extreme climate scenarios (RCP_8.5_); but, by 2100, climate drivers generally outweighed deposition. While more than 70% of counties showed reductions in aboveground carbon relative to the reference scenario, these were not evenly distributed across the US. Counties in the Northwest and Northern Great Plains, and the northern parts of New England and the Midwest, primarily showed positive responses, while counties in the Southeast showed negative responses. Counties with greater initial biomass showed less negative responses to climate change while those which exhibited the greatest change in composition (>15%) had a 95% chance of losing carbon relative to a no-climate change scenario. This analysis highlights that declines in forest growth and survival due to increases in mean temperature and reductions in atmospheric N deposition are likely to outweigh positive impacts of reduced S deposition and potential increases in precipitation. These effects vary at the regional and county level, however, so forest managers must consider local rather than national dynamics to maximize forest carbon sinks in the future.

## 1. Introduction

Forests, and the services they provide, are sensitive to environmental stressors including temperature, water and nutrient availability, atmospheric deposition, extreme events, and other ecological conditions. Current trajectories for environmental change show some of these stressors worsening, such as warmer temperatures and varying precipitation, and some improving, such as nitrogen (N) and sulfur (S) deposition. Indeed, average global surface temperatures have been steadily increasing over the past century alongside highly regional changes in precipitation (Hayhoe et al 2018). Meanwhile, nitrogen and sulfur deposition have been decreasing in the US since the 1990s (Lloret and Valiela 2016, EPA 2020). The expected impacts on forests will depend upon various factors including edaphic conditions, species composition, and the presence or absence of key symbionts (e.g. mycorrhizal fungi) and secondary stressors (e.g. pests). Therefore, global or national-extent analyses can obscure forest responses to environmental change at scales relevant to forest management. Instead, county or regional analyses can identify areas where climate change and deposition reduction are most likely to promote or undercut beneficial ecosystem services like carbon sequestration.

Climate change due to anthropogenic greenhouse gas emissions will result in an array of physical and chemical changes across the United States. Average temperature increases are the most frequently considered but changes in precipitation timing and intensity, concentrations of CO_2_, and extreme events, amongst others, are all also anticipated (Hayhoe et al 2018). Each will manifest to varying degrees in different regions. For example, higher latitudes have warmed faster than lower latitudes, while the parts of the Southeast have warmed less than half as quickly as other areas in the US (Marvel et al 2023). All are anticipated to be consequential for forest composition and functioning, with direct effects, and interactive effects between local changes in temperature, precipitation, and CO_2_ concentrations leading to geographic variation in forest responses. Some impacts are already apparent or locked in for long-lived species (Vose et al 2018) and overall are anticipated to undermine forest ecosystem services (Domke et al 2023a, 2023b). Individual tree growth and survival is mediated by local temperature and precipitation through effects on growing season length and conditions, water stress, and nutrient availability, as well as by CO_2_ levels and disturbance. But, ultimately, the impact of climate change on forests will vary based on starting physical and biological conditions in a given forest or region (Charney et al 2016, Xu et al 2020, Gao et al 2022, Clark et al 2023a) as well as the local manifestation of climate change.

The deposition of reactive N and S has dramatically increased since pre-industrial times due to anthropogenic activity. Across the United States, minimum critical loads are fairly uniform and have been consistently exceeded since the early 20th century (Clark et al 2018). Exceedances of critical loads peaked in the 1970s and 1980s and current EPA policies could lead to a continued decrease through the coming decades (Clark et al 2018). Although there are signs of recovery (Lawrence et al 2015, Hazlett et al 2020), it is unclear how quickly or how widespread ecological recovery will be (Gilliam et al 2019). Climate change may also have an impact on deposition trends, although these are anticipated to be less than emission reduction impacts (Tagaris et al 2008, Clark et al 2018). Ultimately, both N and S can have acidifying effects in terrestrial ecosystems (EPA 2020), but N enrichment can also lead to increased primary productivity by eliminating nutrient limitation (Elser et al 2007, Quinn Thomas et al 2010). Differential sensitivity to N and S deposition can lead to altered competitive balance of species and thus changes in biodiversity and forest composition (Horn et al 2018, Clark et al 2023a, Coughlin et al 2023). The impact of deposition varies widely across the country depending on the presence or absence of sensitive species, as well as local conditions (EPA 2011). Similarly, anticipated reductions vary based on regional actions (EPA 2011). Notably, N deposition in the eastern United States and S deposition in the Ohio Valley and mid-Atlantic regions are consistently more pronounced than elsewhere in the country (Benish et al 2022). Therefore, local or regional responses to changing deposition patterns will reflect both starting community composition and physical deposition trends.

This work is part of a large multi-year effort by the U.S. Environmental Protection Agency to better understand how forest communities may be impacted by changes in climate and atmospheric deposition (Phelan et al 2016, Van Houtven et al 2019, Le Duc et al 2022, Clark et al 2023a, 2023b, Coughlin et al 2023). Similar to other studies (Van Houtven et al 2019), a single cohort of trees was modeled, and only mean annual temperature and mean annual precipitation were included as climate predictors in our assessments (limitations of this discussed below).

## 2. Methods

### 2.1. Overview

Here, we investigated sub-national forest aboveground carbon dynamics for the conterminous US through 2100 using the previously developed simple forest model (Van Houtven et al 2019, Clark et al 2023a). We modeled growth and survival of existing trees under five climate change trajectories crossed with four deposition scenarios (20 total climate-deposition scenarios; figure S1). The projections leverage a recent assessment of species-specific growth and survival models for 94 species, representing the vast majority of trees in the forested areas of the conterminous US (Horn et al 2018). Those models in Horn et al (2018) capture species responses to mean annual temperature, mean total precipitation, S deposition, N deposition, tree size, and stand competition, and were applied to the U.S. Forest Services (USFS) Forest Inventory and Analysis (FIA) 2000–2016 tree database as the starting cohort (Clark et al 2023a). Clark et al (2023a) examined how each of the 94 species are affected by changes in N deposition, S deposition, temperature, and precipitation; and, how that affects compositional difference of the community (as the % overlap in the relative biomass of the tree community). This effort examines how these species level changes add up to influence aboveground forest carbon in the near term (2010–2050) and longer term (2010–2100) horizon, and which factors most influence those carbon responses.

Our model is not a dynamic process model, it is an empirical model of forest carbon based on the observed species-level growth and survival response curves in Horn et al (2018). Thus, it should not be interpreted as a projection of the future, where many non-modeled factors that also affect forests and will change; rather, it is an empirical estimate of forest carbon holding all non-modeled factors (e.g., fire, pests) constant at current levels to isolate the effects of temperature, precipitation, and atmospheric deposition of N and S.

### 2.2. Forest model

We used a set of published projection model results (Clark et al 2023a) as the starting point of our analyses. A full description of the modeling approach is available in the Supplementary Material. Briefly, species-specific growth and survival equations published in (Horn et al 2018) were applied to trees in an initial database, drawn from the FIA program. Only trees with stems ⩾12.7 cm at 1.3 m height from species with at least 2000 records (i.e. 94 of the 352 tree species in the database) were included. This cohort represented 93.2% of plot basal area. Tree numbers were expanded to the county-level, using FIA tree- and plot-specific expansion factors and equations (O’Connell et al 2017). Aboveground biomass, reflecting individual tree growth and survival, was modeled each decade from 2010 to 2100 as a function of six predictors: temperature, precipitation, N deposition, S deposition, tree size, and competition. Aboveground carbon (kg) was calculated as 50% of total biomass (Horn et al 2018) for all trees in a county. Changes in forest composition were estimated as in Clark et al (2023a), which is a percent difference index for relative abundance of all species in a county (see SI for more information).

Climate and deposition parameters were drawn from a suite of scenarios representing potential pathways for the coming decades. The four deposition scenarios (figure S1) included constant deposition (D_0_), N deposition reduction scenario (D_N_), S deposition reduction scenario (D_S_), and N and S deposition reduction scenario (D_NS_). The five climate scenarios (figure S1) included current climate and four future climate scenarios based on different Earth System Models from two IPCC AR5 Representative Concentration Pathway (RCP) emission scenarios: constant climate scenario (CC), a modest climate change scenario based on RCP4.5 (C_4.5_), a moderate climate change scenario based on RCP8.5 (C_8.5,mod_), and two severe climate change scenarios based on RCP8.5—wetter (C_8.5,wet_) or drier dry (C_8.5,dry_). The term ‘moderate’ here does not mean moderate levels of climate change, as RCP8.5 is the most severe emissions scenario examined in the CMIP5. Here ‘moderate’ means a moderate *scenario* among the set of scenarios examined, as there is one that has less climate change (RCP4.5) and two that have more (RCP8.5_wet_ and RCP8.5_dry_, figure S1). Also note that temperature changes under RCP4.5 still exceed the 1.5 degree Paris Agreement, though to a lesser extent than RCP8.5. Total annual N and S deposition, annual precipitation, and average annual temperature were averaged over 10 year periods (2010–2019….2090–2099). These decadal averages were used to drive the changes in growth and survival from 2010–2100 using the best models from Horn et al (2018) for each of the 20 scenarios. For analyses here, we primarily focused on two projection windows: through 2050 and through 2100. Regional variation in the scenarios for 2100 is shown in the SI (figures S6 and S7).

### 2.3. Statistical analyses

We calculated the relative difference between a given model scenario and the reference scenario for each time point. The *reference scenario* was chosen based on first principles and defined as a future with constant 2010 climate conditions and reduced N + S deposition (D_NS_/CC). Continuing deposition reduction is relatively certain and is expected to occur over the next few decades (Clark et al 2023a, EPA 2023). In addition, for scenarios with constant deposition, we calculated the relative difference between a given model scenario and the *baseline scenario*, defined as constant 2010 climate and deposition (D_0_/CC). This relativization method was used for analyses of the relationship between composition change and carbon storage change, as the composition change measurements had been relativized in the same manner (Clark et al 2023a). All other analyses are relative to the reference case scenario to allow for easier comparison between counties which differed in size and baseline forest biomass.

Because the climate and deposition drivers are simultaneously acting on the accumulation of carbon in the underlying growth and survival curves (Horn et al 2018), and these drivers themselves are changing through time and across scenarios, we ran post-hoc statistical analyses to isolate the effects of each driver on forest aboveground carbon. We focused on two time periods, 2050 and 2100. Note that our assumption of a linear ramp for climate scenarios may somewhat underestimate the effects from RCP4.5 as temperature is predicted to increase more in the earlier part of the century than later. For this we used a set of linear mixed effects models (LMEMs) to characterize the importance of model scenarios, time, and geographic variation. First, we ran a LMEM which included model scenario (19 total) and time point (through 2050 or through 2100) as additive fixed effects. For random effects the model included county nested within NCA region. This gave us an estimate of the strength of effect for each model scenario while controlling for time and geographic variation. Second, we were interested in how the dominant drivers (e.g. deposition vs. climate) may change through time. For this we ran two LMEMs, one for each time point (2050 and 2100), which included climate scenario (4 total) and deposition scenario (3 total) as additive fixed effects. For random effects it again included county nested within NCA region. Finally, we were interested whether the climate responses by NCA region differed for different drivers and time periods. For this analysis only the subset of models with N and S deposition reduction were used, we ran two LMEMs, one for each time point, which included final temperature, final precipitation, and region as interactive fixed effects and county as the random effect.

All LMEMs were implemented using the lme4 package (Bates et al 2015) in R (version 4.2.2). We used the lmerTest package (Kuznetsova et al 2017) to calculate *p* values for ANOVAs based on Satterhwaite’s method. We also calculated estimated effect sizes (d), a unitless term, for each fixed effect as the estimate for the fixed effect divided by the square root of the sum of variances for random effects (Westfall et al 2014, Brysbaert and Stevens 2018). Effect sizes were deemed small (0.2 ⩽ |*d*|< 0.5), medium (0.5 ⩽ |*d*| < 0.8), or large (|*d*| ⩾ 0.8) (Carson 2012). These effects are relative to an instance of each categorical fixed effect variable: for scenarios, that instance is the D_NS_/CC scenario; for climate, that instance is the CC scenario; for deposition; that instance is the D_NS_ scenario; and for region, that instance is the Northeast (the area of focus for a previous modeling study (Van Houtven et al 2019)).

We also conducted a series of Spearman correlation analyses. First, we tested for a correlation between 2050 carbon responses and 2100 carbon responses. For the subset of models with N and S deposition reduction, we also tested for a correlation between county baseline biomass and carbon responses. Finally, for the subset of models with constant deposition and one of three climate scenarios (C_4.5_, C_8.5,wet_, or C_8.5,dry_), we tested for a correlation between carbon responses and forest composition responses (as calculated in (Clark et al 2023a)). All correlations were carried out using the ‘r.corr’ function in the R package Hmisc (Harrell Jr and Harrell Jr 2019).

## 3. Results

**Figure 1.**
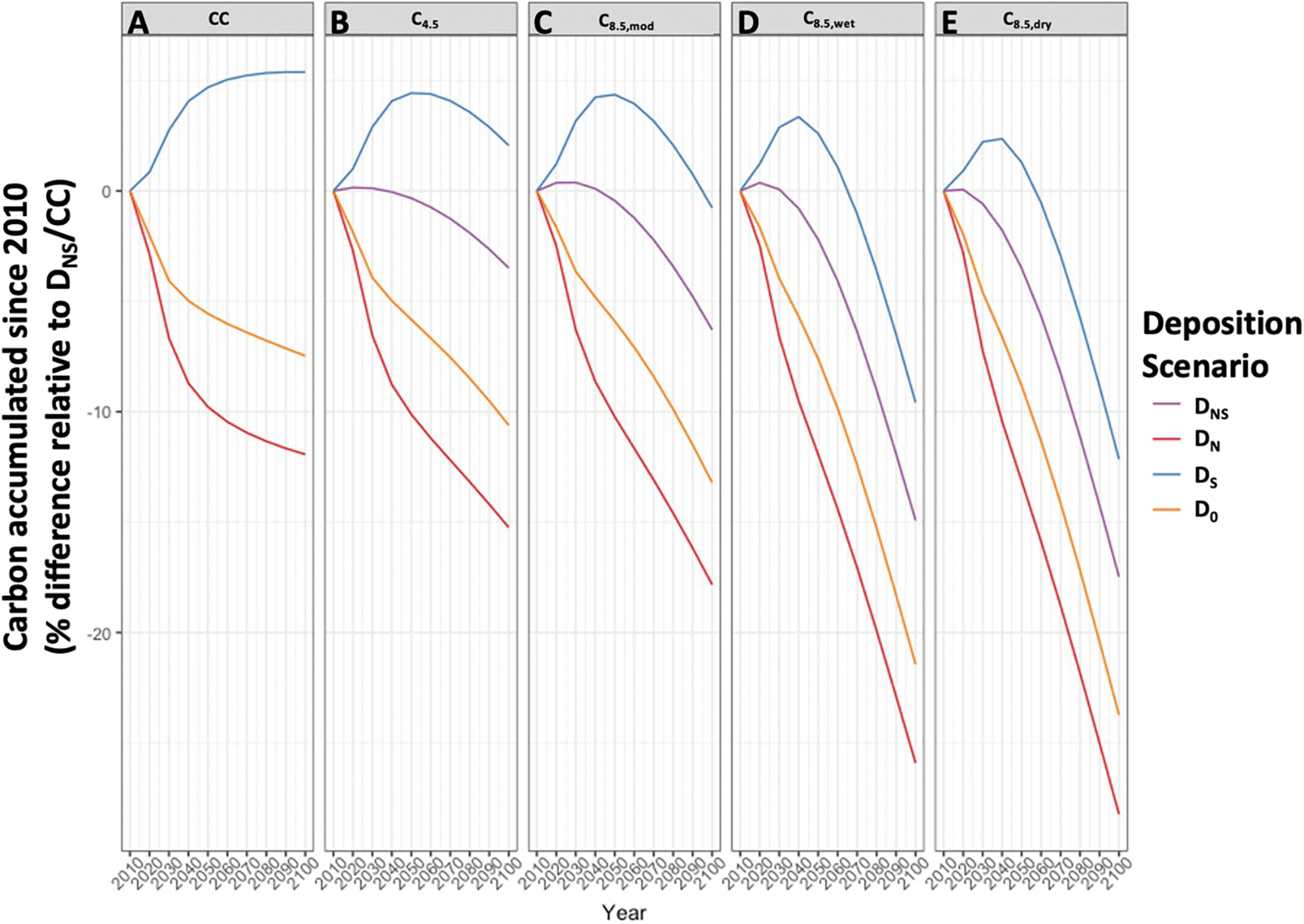
Projected new carbon accumulation in aboveground biomass of existing cohort of trees through 2100, summed across the contiguous United States. Panels A-E shows climate scenarios, and lines within panels are for deposition scenarios. Carbon accumulation is relative to the reference scenario in 2010 (D_NS_/CC).

We found that future aboveground carbon in the current cohort will respond significantly to climate and deposition changes, but the effects will vary across time, space, and scenario. Nationally, aboveground carbon is predicted to accumulate in the cohort of trees through the twenty-first century (figure S2), but climate change and continued atmospheric S deposition will dampen this accumulation compared to our reference scenario (D_NS_/CC; figure 1). Only scenarios with continued N deposition, but reduced S deposition, and mild or no climate change have higher national aboveground carbon than the reference by end of century. Ever increasing temperature in our scenarios (C_4.5_ < C_8.5,mod_ < C_8.5,wet_ ~ C_8.5,dry_) led to greater and greater reductions in aboveground carbon accumulation, with little effect of differences in precipitation (C_8.5,wet_ versus C_8.5,dry_, figure 1).

**Figure 2.**
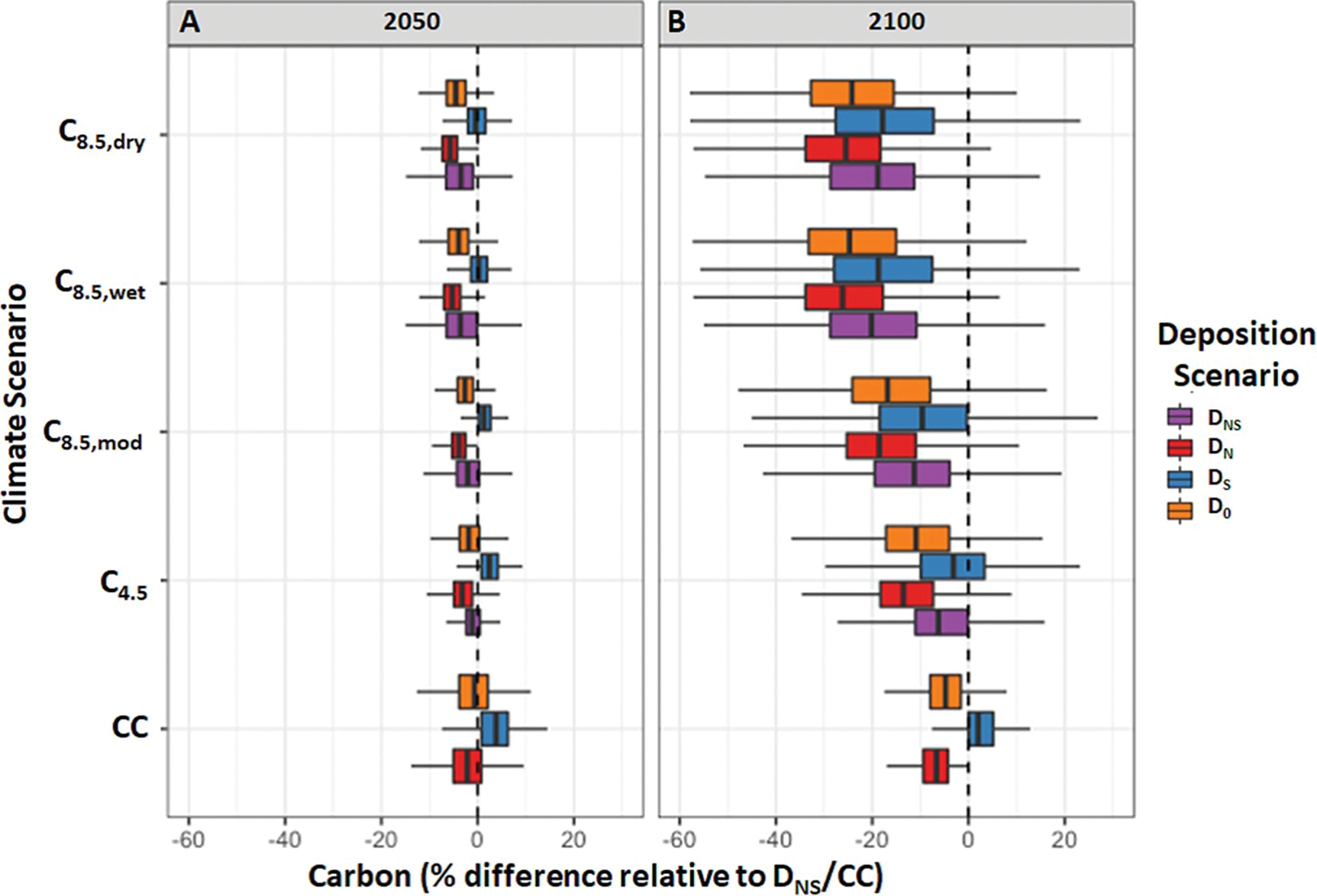
Projected county-level aboveground forest carbon responses in 2050 (A) and 2100 (B). Climate scenarios are on the *y*-axis and colors indicate deposition scenarios. Response values are relative to the reference scenario (D_NS_/CC). Box plots show median, first and third quartiles, calculated minima and maxima.

Across the full suite of simulations, we found the accumulated aboveground carbon pools at the county level differed significantly among scenarios (*p* < 0.001, ANOVAs of LMEM fits) compared to our reference scenario (figure 2). As seen nationally, more extreme climate change generally led to further decreasing aboveground carbon, while reduction in N deposition tended to reduce aboveground carbon and reduction in S deposition tended to increase aboveground carbon (figure 2). On a net basis, reductions in both N and S deposition tended to increase aboveground carbon, suggesting the deleterious acidifying effect of S has a larger impact compared to the loss of the fertilizing effect of N under a cleaner air scenario. These effects were offset by midcentury even under scenario C_4.5_ (figures 1 and 2). Aggregate scenario effect sizes were typically small to medium except for cases with very hot climate conditions which had large effect sizes (table 1). For all but the constant climate scenarios, the negative effect from climate change outweighed the positive effect from continued N deposition for mid- and late-century timepoints, indicating mean effects are negative for all but one scenario (*i.e.*, D_S_/CC, figure 2, table 1).

Time also had a large effect. Overall, impacts of climate and deposition change were much larger later in the century, and the variance in responses among counties was also larger (figure 2(B)). Analyzing results for each time period (through 2050 and through 2100) separately, we found climate and deposition terms were significant in both models (*p* < 0.001, ANOVAs of LMEM fits, table 2), and the county-level responses in mid- and late-century were correlated (*p* < 0.001, *ρ* = 0.59, Spearman correlation; figure S3). Interestingly, when we compare mid- and late-century, we found the importance of scenarios was different for the two time periods. In mid-century, deposition scenarios had comparable effect sizes in absolute terms to climate scenarios (table 2), whereas later in the century, the climate scenarios, particularly the RCP8.5 scenarios, had much larger effect sizes than the deposition terms. While the absolute importance varies, the relative effect of N and S deposition reduction, as well as their additive impact in combination, was consistent for both time periods.

To unpack regional and county-level variation, we focused on the subset of simulations with reductions in both N and S deposition (D_NS_). Relative to no change in atmospheric deposition, the D_NS_ scenario typically led to greater aboveground carbon, although the effect size was smaller than any climate term (table 2). Hereafter, we focus on D_NS_ because—although future changes in climate remain uncertain—continuing reductions in N and S deposition are relatively certain and expected to occur over the next few decades (EPA 2023). Figure summaries of the full results at the regional scale are available in the supplement (figures S4 and S5).

**Figure 3.**
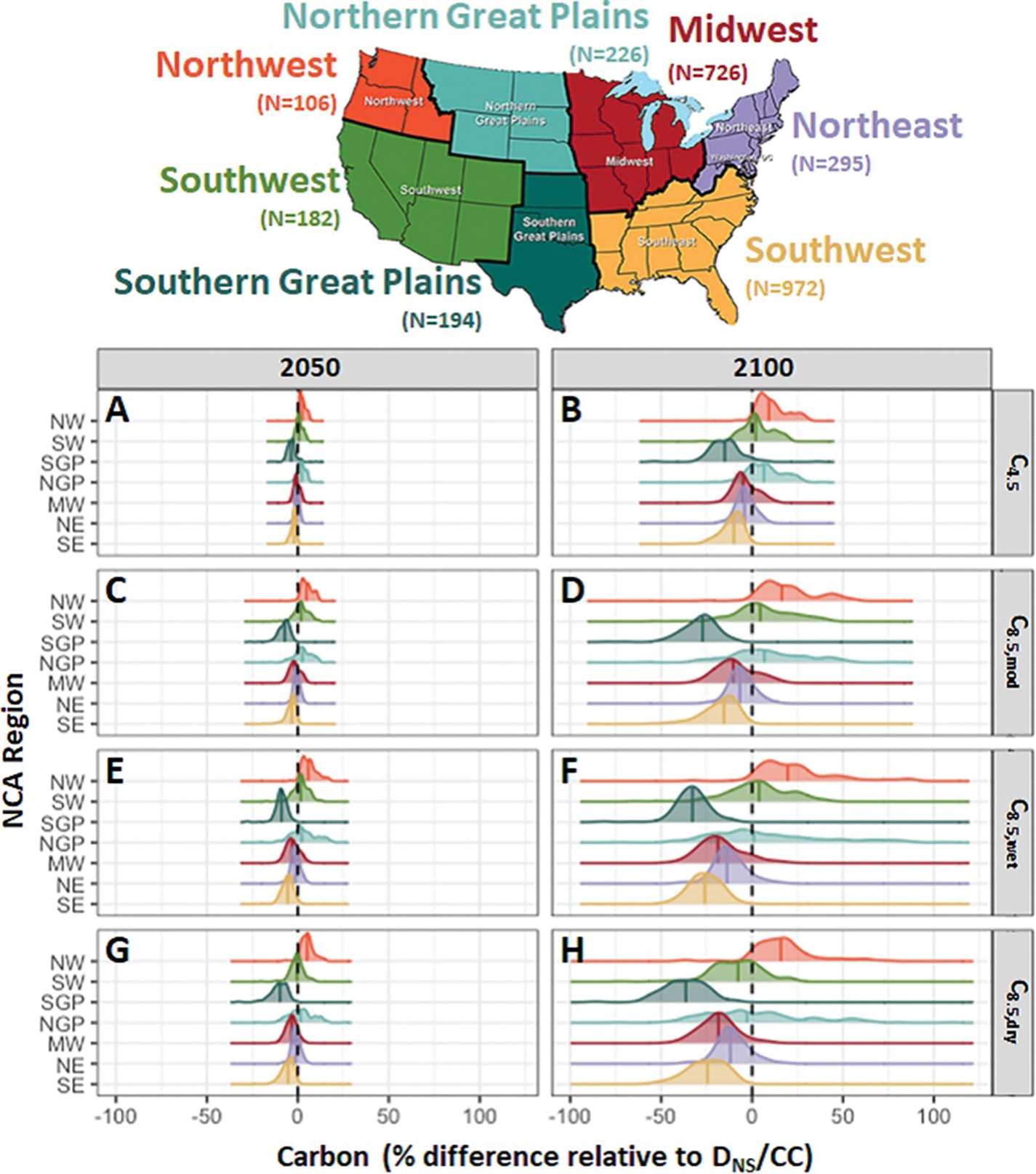
Density plots of projected county-level aboveground forest carbon responses to varying climate scenarios in 2050 (A, C, E, G) and 2100 (B, D, F, H) relative to the reference (D_NS_/CC) scenario. *Y*- axis and colors indicate USGCRP National Climate Assessment regions: Northwest (NW), Southwest (SW), Southern Great Plains (SGP), Northern Great Plains (NGP), Midwest (MW), Northeast (NE), and Southeast (SE). Solid vertical lines indicate regional median. Only D_NS_ scenarios are plotted (for all scenarios see figures S4 and S5).

**Figure 4.**
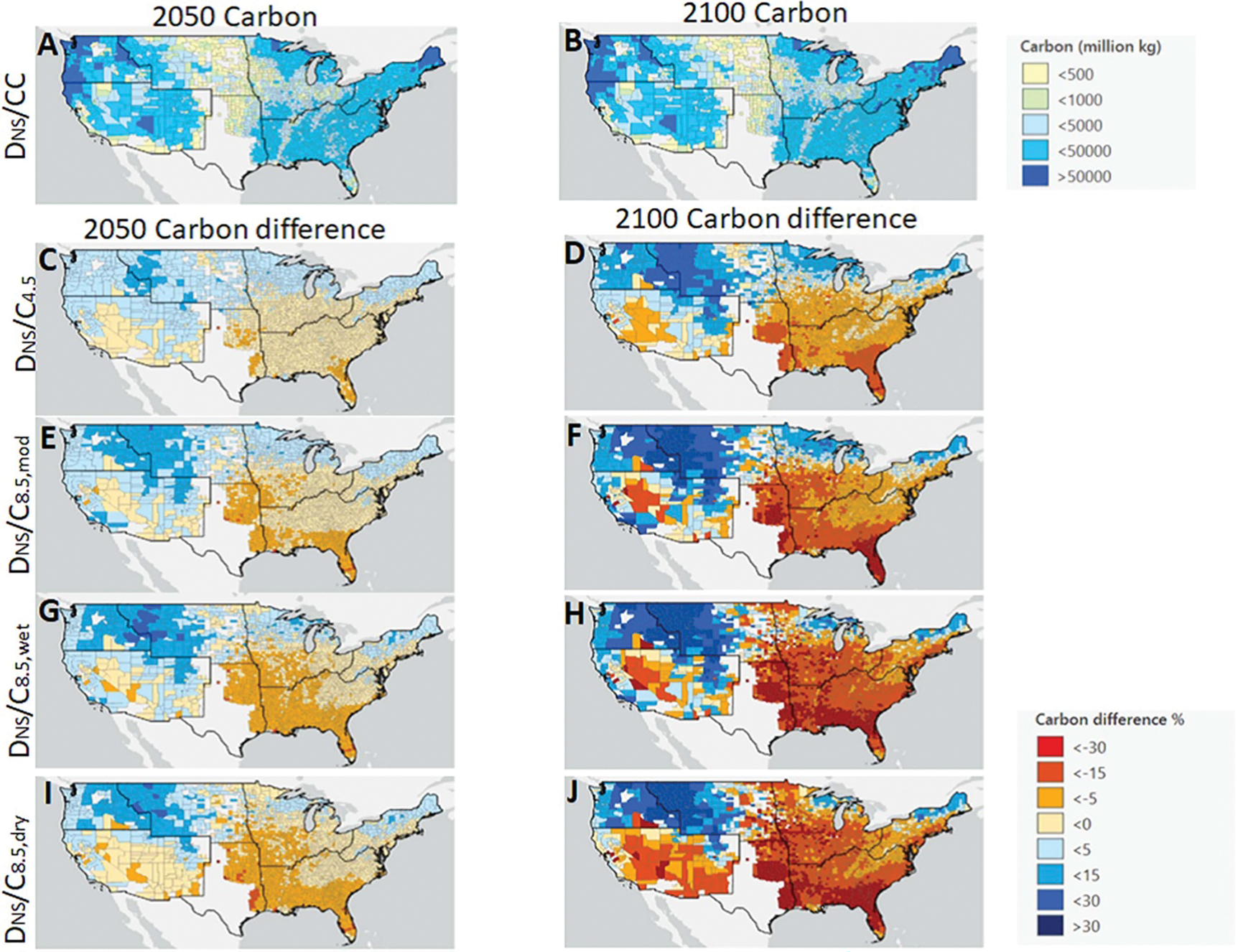
Projected county-level aboveground forest carbon (million kg C) under D_NS_/CC scenario in 2050 (A) and 2100 (B). Projected county-level aboveground forest carbon responses under varying climate scenarios for 2050 (C), (E), (G), (I) and 2100 (D), (F), (H), (J) as per cent difference from D_NS_/CC scenario. Black lines delineate USGCRP National Climate Assessment regions (note, white counties are areas with no FIA plots that are characterized as forests).

Aboveground carbon responses to climate change varied widely at a regional (figure 3) and county-level scale (figure 4). Under RCP4.5, which is consistent with a 1° C–3° C increase depending on the region (figure S6), we found that at the end of century 75% of counties had decreases in aboveground carbon relative to our reference scenario (figure 3). The Southern Great Plains, Midwest, Northeast, and Southeast regions exhibited decreases, whereas the Northwest and Northern Great Plains increased. Decreases in carbon corresponded with the eastern areas that were already warmer (e.g. southerly) and were projected to warm further. The median effect in the Southwest region was near zero. Under RCP8.5 scenarios, 83% of counties showed decreases in aboveground carbon relative to the reference case. The same regional patterns persist as under RCP4.5, although the effects were larger. Differences in precipitation between RCP8.5_wet_ and RCP8.5_dry_ were not projected to alter regional patterns, except in portions of the Southwest (e.g. Southern California, New Mexico), where the drying under C_8.5,dry_ was most pronounced (figure S6). Here, carbon increased under wetter conditions and decreased under drier conditions (e.g. median of +5% and −8% in 2100, respectively, for SW in figure 3(F) vs. (H); and figure 4(H) vs. (J)). Temperature and precipitation terms were significant in LMEMs of county-level variation (*p* < 0.001, ANOVAs of LMEM fits; table S1), but their effect sizes were smaller than those for the regional terms and some of the interactions between regional terms and the climate parameters. As noted for CONUS-wide results, effects in 2050 were similar in direction to those in 2100, although there were slightly fewer decreases in carbon (70% and 76% under RCP4.5 and RCP8.5, respectively) and the magnitude of the differences was generally smaller.

**Figure 5.**
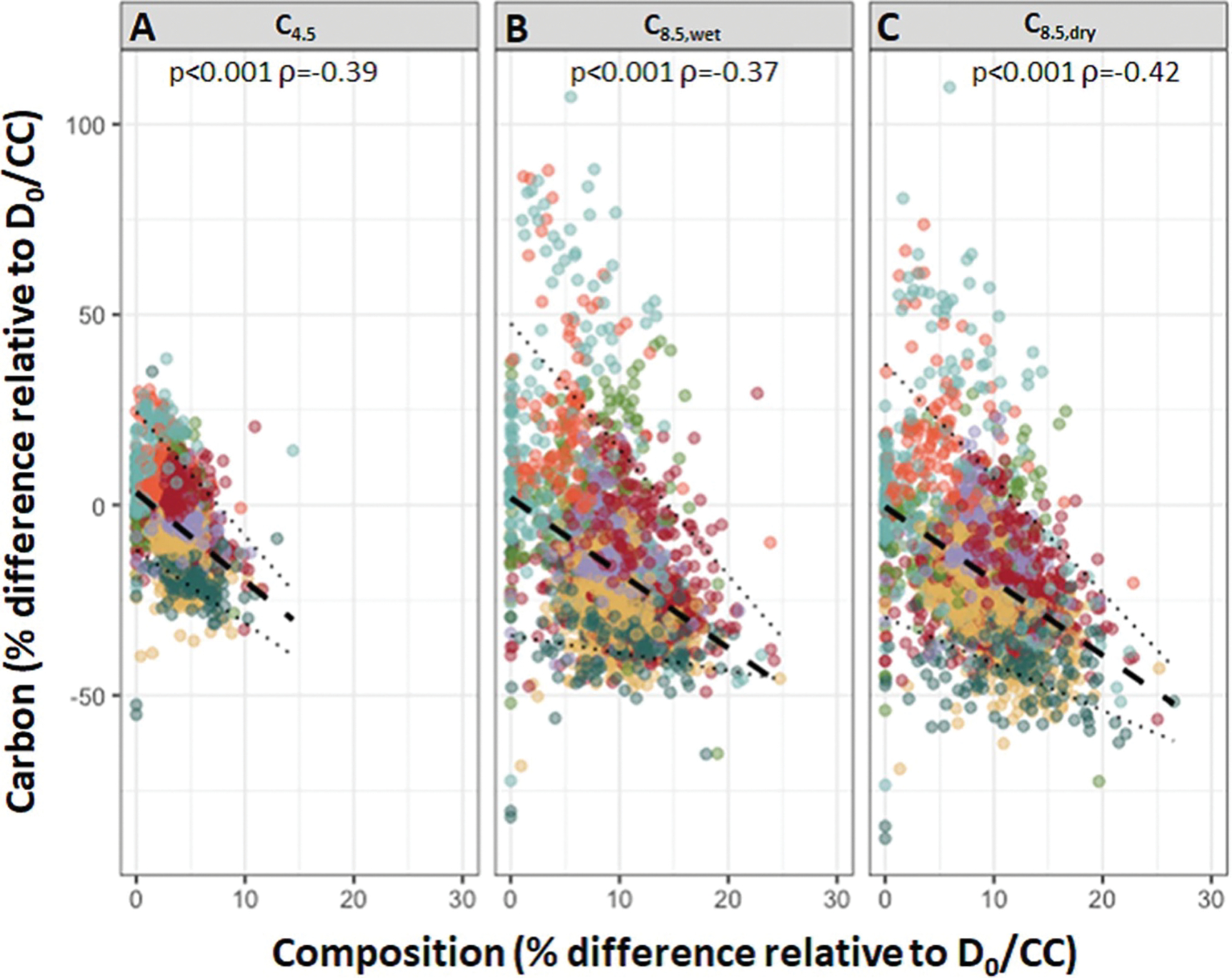
Projected county-level aboveground forest carbon response in 2100 as a function of county-level forest composition change. Panels indicate climate scenario with response calculated relative to D_0_/CC scenario for C_4.5_ (A), C_8.5,wet_ (B), and C_8.5,dry_ (C). Dashed lines indicate linear fit and dotted lines indicate 95% confidence interval for the fit. Spearman correlation results are inset for each panel. Colors indicate USGCRP National Climate Assessment regions as in figure 3

We also tested for relationships between aboveground carbon response and some key forest characteristics at the county-scale. Counties with greater starting biomass tended to have a less negative response to climate change (2100 *p* < 0.001, *ρ* = 0.27; 2050 *p* < 0.001, *ρ* = 0.25 Spearman correlation). County level carbon responses were also negatively correlated with the amount of compositional change experienced (*p* < 0.001, *ρ* < −0.37, all scenarios; figure 5). All plots experiencing large compositional change had large reductions in carbon compared to the baseline scenario (D_0_/CC), but some counties exhibited similar reductions in carbon without similar compositional change. With a 15% or greater change in composition, there was a 95% chance of decreased carbon relative to the baseline scenario.

## 4. Discussion

Forests currently serve as a net greenhouse gas sink in the U.S., representing the largest component of land sector carbon sequestration (Domke et al 2023b). However, diminished tree growth and survival under global change anticipated in the coming decades may undermine forests critical role in climate mitigation (Ohrel 2019). Using a simple forest dynamic model to investigate carbon dynamics, we find most forests in the conterminous US will accumulate less aboveground carbon in a future with more extreme climate change or with a failure to reduce acidic deposition, all else held constant. However, these responses vary significantly between and within regions, with implications for local land management strategies and emerging carbon markets.

This paper focuses on aboveground forest carbon. For a detailed discussion of the underlying growth and survival relationships see Horn et al (2018) and for a detailed discussion of how these relationships were translated to changes in forest composition see Clark et al (2023a). Generally, the growth and survival relationships fit the data relatively well (see SI, *R*^2^ for growth models ranged from 6% to 51%, Horn et al 2018). The projected changes in forest composition are difficult to validate since most experimental work is only on a handful of the 94 species assessed here, and FIA full-panel data only go back a few decades. However, with some exceptions our compositional projections generally agreed with expectations (Clark et al 2023a). For example, in an experimental study on juvenile trees, Reich et al (2022) found that increases in temperature (+1.6 °C and +3.1 °C) comparable to our RCP4.5 simulations led to increased growth for *Acer rubrum* and *Acer saccharum* and decreased growth for *Abies balsamea, Picea glauca,* and *Pinus strobus*. Our results generally agree, with the RCP4.5 scenario predicting an increase in Acer rubrum (1.5%) and Acer saccharum (7.7%), and a decrease in Abies balsamea (−10.2%) and Picea glauca (−16.9%) (Clark et al 2023a). An observational study in the western United States that also used the FIA data (Stanke et al 2021), found that *Pseudotsuga menziesii* and *Pinus ponderosa* were increasing while *Abies lasiocarpa, Populus tremuloides, Picea engelmannii,* and *Pinus contorta* were decreasing. Direct comparisons with Stanke et al (2021) are difficult as the measures of abundance are very different between the studies, but we also found *Pseudotsuga menziesii* and *Pinus ponderosa* were projected to increase mostly from positive effects of increases in temperature (Clark et al 2023a).

Under the RCP4.5 scenario, representing the 1.5 °C–2 °C world most likely representative of the coming decades, we found that overall forests will show small but significant reductions in aboveground carbon relative to a no climate change future, even with cleaner air from reduced deposition of N and S. Much of the northern United States may actually benefit under a RCP4.5 future, but forests in the Southeast and Southern Great Plains regions delineated by USGCRP, as well as the lower Midwest and Southwest may see reductions in excess of 10%. Within the regions, there is further variation. Under more extreme climate change (RCP8.5 scenarios), there are greater carbon reductions predicted, but even then, at least 15% of counties are predicted to have more carbon under global change scenarios compared to the reference scenario. Again, these counties are primarily found in the Northwest and Northern Great Plains regions and the upper Midwest and Northeast, typically in areas with larger baseline biomass. These could be high priority areas for future forest protection and afforestation to support the land carbon sink. In a recent study on the timber industry using the same underlying curves from Horn et al (2018) and using a dynamic forestry model (FASOM), Baker et al (2023) found that there could be a massive relocation of the timber industry from the southeast to the northwest under future climate scenarios, highlighting the likelihood of conflict between potential forest services.

More detailed biogeochemical studies such as in the Harvard Forest (Melillo et al 2017) have documented distinct phases in soil respiration as more and more recalcitrant soil carbon pools are mineralized following changes in the soil microbial community. These could impact N availability to the trees and thus our projections. Repeat analyses like ours of the trees using multiple periods could elucidate whether this phenomenon is widespread, and which forests in the U.S. are in which phase.

Across regions, changes in forest composition were generally an effective predictor for carbon outcomes in our models. Counties with larger shifts in community composition were almost certain to have negative carbon responses. However, counties with smaller shifts in community composition were as likely to have increased carbon as decreased relative to the baseline scenario. This result suggests some forests may appear similar in the future while providing very different carbon sequestration and perhaps other ecosystem services.

Unsurprisingly, we predict bigger deviations in forest carbon to appear later in the century when the magnitude of change in drivers is larger and there has been more time for impacts to accumulate. The reductions in carbon predicted under more stressed scenarios at the end of the 21st century primarily reflect forest responses to climate parameters. In contrast, deposition generally matters more than climate in mid-century. Among the climate change scenarios, we find the effect sizes associated with temperature and precipitation changes are similar to one another in each time period (table S1), demonstrating the importance of both for driving responses to persistent climate change.

Additional mortality due to climate change driven increases in pulse disturbances, including wildfires, insect outbreaks, and extreme weather events, are also anticipated to contribute to changes in forest carbon in a highly localized manner (Berner et al 2017, Kim et al 2017, Anderegg et al 2022). These are not directly captured in our models here. While current mortality parameters in our model capture differential disturbance effects observed in the parametrization period (2000–2016), further anticipated changes to disturbance regimes, such as the anticipated growing wildfire impacts in the western US (Vose et al 2018), are absent from our projections, probably resulting in us overestimating local and national carbon levels under climate change scenarios. A recent synthesis projected 45%–65% less aboveground carbon nationally in wildfire and climate stressed scenarios compared to a no stress/disturbance case; those losses could largely offset or outweigh potential growth benefits in the northern US (Wu et al 2023).

Our study also does not include the potential for elevated CO_2_ to stimulate tree growth, improve water use efficiency, and offset a portion of the decreases projected here (Song et al 2019, Chen et al 2022, Davis et al 2022). CO_2_ fertilization may also aggravate N limitation (Luo et al 2004), which would be exacerbated in the D_N_ scenario. It was not possible to incorporate the potential CO_2_ fertilization directly in our analysis given the spatial differences in CO_2_ are small and the FIA plots have only been resampled in the past few decades. Furthermore, the strength and longevity of the CO_2_ fertilization effect is still uncertain and actively debated, with no effect or little effect in some studies (Gedalof and Berg 2010, Girardin et al 2016), and strong effects in highly controlled studies (Song et al 2019). In combination with local variation in atmospheric deposition, temperature, and precipitation, however, the effects of elevated CO_2_ concentrations will likely lead to further deviations in forest composition and productivity in decades to come.

We present our findings throughout as relative to a reference scenario (D_NS_/CC) rather than as an absolute change in carbon stored in forests. The reason we do this is because there is no recruitment of new trees yet in this modeling architecture. There are some studies that have modeled recruitment in the eastern U.S. using similar architecture (Canham et al 2016), but none have linked this to the addition of new adult trees, nor extended these to the CONUS. Until these limitations are overcome our results are best interpreted as changes forest carbon from the individual drivers examined—holding all else constant—from the current cohort of trees through time, rather than changes in U.S. forests carbon through time from all factors. That said, the current cohort represents trees of a wide range of size and age classes, and probably represents most of the U.S. forests over much of this century. Relativizing to a reference scenario allows for direct comparison of trajectories between counties and regions by adjusting for differences in baseline forest characteristics and size of counties. Nonetheless, because of these limitations, our results do not indicate whether forests are net sinks or sources of greenhouse gases in the future, but only the relative sensitivity of the current forest cohort to a given future scenario at specific points in time. Overall, we anticipate global change will reduce forests’ capacity to mitigate climate change by accumulating less carbon compared to current climatic conditions. Some recent analyses, including the recent USFS Resource Planning Assessment, have predicted U.S. forest will become a net carbon source later this century due to climate change induced mortality and increasing wood product demand (e.g. Wear and Coulston 2015, USFS 2023), but others merely predict a slowdown in carbon gains (e.g. Wu et al 2023). The reductions in aboveground carbon in our projections are not inconsistent with either but cannot be directly compared.

## 5. Conclusion

Differential sensitivity to global change parameters between species and across the landscape will result in fine-scale variation in forest composition and function in the coming decades. Our demographic model illustrates potential effects of changing average temperature, precipitation, and the atmospheric deposition of N and S on aboveground carbon, finding a general decrease in this critical ecosystem service but some regions would perform better under future regimes. This finding complements other recent analyses showing regional and local variation in forest carbon depending on intermittent climate stressors (e.g. Wu et al 2023), and it accentuates the need for spatial resolution in projection modeling efforts. Resource managers will need to consider how their particular forests are likely to respond, rather than national averages, to increase the likelihood of successful adaptation or promote nature-based climate solutions.

## Supplementary Material

Supplement1

## Figures and Tables

**Table 1.** Results of ANOVAs of linear mixed effects model for projected aboveground forest carbon response as a function of projection scenario and year, as fixed effects, and county nested in NCA region, as random effects. All *p* values were less than 0.001. Model estimate and estimated effect size (*d*) for each fixed effect term are recorded. Effect size cells are colored with yellow indicating small effects (0.4 > |*d*| ⩾ 0.2), orange indicating medium effects (0.8 > |*d*| ⩾ 0.4), and red indicating large effects (|*d*| ⩾ 0.8).

Variable	Estimate	Effect size (*d*)

Year	−10.02	−0.80
D_0_/CC	−2.685	−0.21
D_N_/CC	−4.606	−0.37
D_S_/CC	3.323	0.27
D_0_/C_4.5_	−8.361	−0.67
D_N_/ C_4.5_	−10.15	−0.81
D_S_/ C_4.5_	−2.641	−0.21
D_NS_/ C_4.5_	−5.677	−0.45
D_0_/C_8.5,mod_	−5.775	−0.46
D_N_ /C_8.5,mod_	−7.634	−0.61
D_NS_/C_8.5,mod_	−3.086	−0.25
D_0_/C_8.5,wet_	−11.99	−0.96
D_N_/C_8.5,wet_	−13.65	−1.09
D_S_/C_8.5,wet_	−6.502	−0.52
D_NS_ /C_8.5,wet_	−9.343	−0.75
D_0_/C_8.5,dry_	−13.1	−1.05
D_N_/C_8.5,dry_	−14.76	−1.18
D_S_/C_8.5,dry_	−7.722	−0.62
D_NS_/C_8.5,dry_	−10.56	−0.84

**Table 2.** Results of ANOVAs of linear mixed effects models for projected aboveground forest carbon response under each time period as a function of climate and deposition scenario terms, as fixed effects, and county nested in NCA region, as random effects. All *p* values were less than 0.001. Model estimate and estimated effect size (d) for each fixed effect term are recorded for the 2050 model (A) and the 2100 model (B). Effect size cells are colored with yellow indicating small effects (0.4 > |*d*| ⩾ 0.2), orange indicating medium effects (0.8 > |*d*| ⩾ 0.4), and red indicating large effects (|*d*| ⩾ 0.8).

Variable	Estimate	Effect size (*d*)

(a)		

C_4.5_	−1.043	−0.28
C_8.5,mod_	−2.043	−0.55
C_8.5,wet_	−3.226	−0.87
C_8.5,dry_	−3.794	−1.02
D_N_	−2.172	−0.58
D_S_	3.523	0.95
D_0_	−0.745	−0.20

(b)		

C_4.5_	−5.1766	−0.41
C_8.5,mod_	−9.3853	−0.75
C_8.5,wet_	−15.5324	−1.24
C_8.5,dry_	−17.2937	−1.38
D_N_	−6.6753	−0.53
D_S_	2.5634	0.20
D_0_	−4.5524	−0.36

## Data Availability

Data are available at the United States government’s Data.gov. The data that support the findings of this study are openly available at the following URL/DOI: http://doi.org/10.23719/1530107.
