# Supplementary material for: Geographic variation in projected US forest aboveground carbon responses to climate change and atmospheric deposition
Source: Environ Res Lett. Author manuscript; Available in PMC 2025 Feb 20. (PMC11091792; doi:10.1088/1748-9326/ad2739)
Supplement: Supplement1 [file NIHMS1987902-supplement-Supplement1.pdf]

Supplementary Material

Supplemental Methods:

Overview of Tree Species Response Curves

Forest Inventory data.

Tree growth, tree survival, and plot-level basal area data were compiled from the Forest Inventory and Analysis (FIA) program database representing the forest cohort from 2000-2016 (accessed on January 24, 2017, FIA phase 2 manual version 6.1; http://www.fia.fs.fed.us/). Tree biomass was estimated from tree DBH measurements multiplied by allometric relationships in (Jenkins et al. 2003) to estimate aboveground biomass. Aboveground biomass is then multiplied by 0.5 to estimate aboveground C. Tree growth rates were calculated from the difference in aboveground C between the latest and first measurement and divided by the elapsed time between measurements to the day. Tree species that had at least 2,000 individual trees after the data filters were applied were retained for both growth and survival analyses. The 1.4 million trees from 94 species modeled in (Horn et al. 2018) represent nearly 100 billion trees in the U.S., representing >90% of trees in most forested areas in the U.S. (Clark et al. 2023). Thus, some important tree species are omitted and require additional study (e.g., Sequoia sempervirens [redwood, <1500 samples]). The probability of tree survival was calculated from the first live measurement to the last live measurement or to the first measurement recorded as dead for each tree inventoried. Trees that were recorded as dead at both measurement inventories and trees that were harvested were excluded from the survival analysis.

Predictor data: Climate, deposition, size, and competition.

There were six predictors that were related to the rate of growth or survival for each individual tree: mean annual temperature (T), mean annual precipitation (P), mean annual total nitrogen deposition (N), mean annual total S deposition (S), tree size (m), and plot-level competition.

To obtain total N and S deposition rates for each tree, we used spatially modeled N and S deposition data from the National Atmospheric Deposition Program's Total Deposition (TDEP) Science Committee (Schwede and Lear 2014). Annual N and S deposition rates were then averaged from the first year of measurement to the last year of measurement for every tree so that each tree had an individualized average N deposition based on the remeasurement years, and each species had an individualized range of average N deposition exposure based on its distribution. At the time of the analysis, deposition from only 2000-2014 was available, so we used the 2014 deposition values for the years 2015 and 2016. Monthly mean temperature and precipitation values were obtained in a gridded (4 x 4 km) format from the PRISM Climate Group at Oregon State (Daly et al. 2008) for the CONUS and averaged between measurement periods for each tree in a similar manner. Tree size was represented by aboveground tree C (m, previously described). Because the climate and deposition predictors were tailored to each plot, the years assessed varied by plot, but spanned 2000-2016. Tree competition was represented by a combination two factors: (1) plot basal area (BA) and the basal area of trees larger than the focal tree being modeled (BAL). How all six variables were statistically modeled is discussed below. Many other factors also influence growth and survival of tree species such as ozone, pests, CO_2 concentrations, and forest management. However, these factors were not included in the original growth and survival

derivations and so were also not included in their application here. We discuss the implications of this and other factors below.

Modeling tree growth and survival.

We assessed in (Horn et al. 2018) multiple models to predict tree growth and survival. Our growth model assumes that there is a potential growth rate (a) that is modified by up to six predictors in our study: temperature, precipitation, N deposition, S deposition, tree size, and competition. The potential full growth model included all six terms (eq. 4 for the general form and eq. 5 for the specific form).

$$G = potentialgrowthrate \times competition \times temperature \times precipitation \times S_{dep} \times N_{dep} \quad \text{(eq. 4)}$$

$$G = a * m^z * e^{(c_1 * BAL + c_2 * \ln(BA))} * e^{-0.5*\left(\frac{ln\left(T/t_1\right)}{t_2}\right)^2} * e^{-0.5*\left(\frac{ln\left(P/p_1\right)}{p_2}\right)^2} * e^{-0.5*\left(\frac{ln\left(N/n_1\right)}{n_2}\right)^2} * e^{-0.5*\left(\frac{ln\left(S/s_1\right)}{s_2}\right)^2}$$
(eq. 5)

To predict growth, we considered each tree species to have an optimal growth rate that was a power function of its size (*m*), where size is in units of aboveground carbon (kg C/tree), *a* is a fitted parameter, and *z* is a fitted parameter. Competition between trees was modeled as a function of plot basal area (*BA*) and the basal area of trees larger than that of the tree of interest (*BAL*) similar to the methods of (Pukkala et al. 2009), where c_1 and c_2 were fitted parameters, and *BA* and *BAL* were observed based on plot conditions. The environmental factors (temperature [*T*], precipitation [*P*], S deposition [*S*], and N deposition [*N*]) were interpolated per the above procedures at the plot location. The effect of the environmental factors on growth were modeled as two-term lognormal functions (e.g., t_1 and t_2 are fitted parameters for the effect of temperature on growth, p_1 and p_2 are two term parameters for the effect of precipitation on growth, etc.). The two-term lognormal functions allowed for flexibility in both the location of the peak (controlled by the value of t_1, p_1, n_1, and s_1), and the steepness of the curve (controlled by t_2, p_2, n_2, and s_2). Thus, in the parlance of critical loads (Pardo et al. 2011) the estimates of n_1 and s_1 are reasonable estimates of the critical loads for N and S, respectively.

We examined a total of six different growth models: (1) a full model with all six terms (eq. 5), (2) the full model but without the N deposition term, (3) the full model but without the S deposition term, (4) the full model but without both N or S deposition terms, and (5) a null model that estimated a single parameter for the mean growth parameter (*a*).

The annual probability of survival (P(s)) was estimated similarly as growth, except that the probability was a function of time and we explored two different representations for competition. The general form of the model is shown in equation 6, and the full survival model in equations 7 and 8 for the two competition forms.

$$P(s) = \left[a \cdot \text{size} \times competition \times temperature \times precipitation \times N_{dep} \times S_{dep}\right]^{time} \quad \text{(eq. 6)}$$

$$P(s) = \left[a * \left[\left(\left(1 - zc_1 e^{-zc_2 * m}\right) * e^{-zc_3 * m^{zc_4}} \right) \left(e^{-br_1 * BA_{ratio}{}^{br2} * BA^{br_3}} \right) \right] * e^{-0.5*\left(\frac{ln\left(T/t_1\right)}{t_2}\right)^2} * \right.$$

$$\left. e^{-0.5*\left(\frac{ln\left(P/p_1\right)}{p_2}\right)^2} * e^{-0.5*\left(\frac{ln\left(N/n_1\right)}{n_2}\right)^2} * e^{-0.5*\left(\frac{ln\left(S/s_1\right)}{s_2}\right)^2} \right]^{time} \quad \text{(eq. 7)}$$

$$P(s) = \left[a * \left(e^{-0.5*\left(\frac{ln\left(m/m_1\right)}{m_2}\right)^2 * -0.5*\left(\frac{ln\left(BA/ba_1\right)}{ba_2}\right)^2 * -0.5*\left(\frac{ln\left(BAL+1/bl_1+1\right)}{bl_2}\right)^2} \right) * e^{-0.5*\left(\frac{ln\left(T/t_1\right)}{t_2}\right)^2} * \right.$$

$$\left. e^{-0.5*\left(\frac{ln\left(P/p_1\right)}{p_2}\right)^2} * e^{-0.5*\left(\frac{ln\left(N/n_1\right)}{n_2}\right)^2} * e^{-0.5*\left(\frac{ln\left(S/s_1\right)}{s_2}\right)^2} \right]^{time} \quad \text{(eq. 8)}$$

A total of nine survival models were examined: four using the formulation for size and competition in eq. 7 (with the same combinations of predictors as above for growth), four using formulation for size and competition in eq. 8, and a null survival model in which a mean annual estimate of survival (*a*) was raised to the exponent of the elapsed time.

Parameters for each of the growth and survival models above were fit for a given species using maximum likelihood estimates through simulated annealing with 100,000 iterations via the likelihood package (v2.1.1) in Program R. Akaike's Information Criteria (AIC) was estimated for all models. We used the best overall model as assessed by AIC. Horn et al. (2018) focused on the "best and most parsimonious model" which meant the model with the least parameters among the set of comparable models (i.e., those within a delta AIC of 2.0 of the best model, (Burnham 2002)). Additional review of all the models in Horn et al. (2018) for this project revealed that for a small number of species, the most parsimonious models resulted in N-only models beating out models with both N and S, and S-only models beating out models with both N and S. Because N and S are often correlated, the N term in the N-only model could absorb some of the statistical information in S deposition and thus the N relationship could shift from positive or unimodal to flat or negative. Opposite shifts could occur for S (i.e., it could become flat or less negative when N was omitted). The degree of shift was related to the degree of correlation between N and S for that species. Because we hypothesize both N and S deposition matter, we decided to use the best overall model as determined by AIC which often was the model with both terms. The variation explained in the models in (Horn et al. 2018) was good for growth (R^2 ranged from 6-51%, averaged 30% for the 94 species +/- 10% standard deviation) and was not reported for survival. Additional details can be found in (Horn et al. 2018).

<u>Application of Response Curves to Estimate Changes in Forest Carbon</u>

Initial tree database

We assembled an initial cohort of live trees for the project. The original database from (Horn et al. 2018) upon which the response curves were derived required *remeasured* trees for estimates of growth and survival (i.e., two or more time points). *Application* of those response curves onto existing trees does not require trees to have been remeasured, thus the dataset used here is an expansion of that in (Horn et al. 2018) including areas with trees that had only been measured once. This database consists of tree- and plot- specific data from the U.S. Forest Service Forest Inventory and Analysis (FIA) program

assembled for the survival models in Horn et al. (2018) but augmented to include all live trees from the most recent measurement between 2000-2016. It includes tree and plot data for 124,431 plots, 352 species, and 2,851,772 individual trees across the conterminous U.S. As per the FIA definition of "tree" and for consistency with (Horn et al. 2018), only stems $\geq$12.7 cm at 1.3 m height are included in the tree database; saplings and trees smaller than 12.7 cm in diameter were not included. Measured trees in the FIA represent a larger population of trees across the landscape through "expansion factors." With the expansion factors, the trees in our initial tree database represent roughly 100 billion trees across the U.S. Tree numbers were expanded to the county-level, using FIA tree- and plot-specific expansion factors and equations (O'Connell et al. 2017). Although the tree data are for conditions measured in 2000-2016, all trees are assumed to be their reported FIA biomass in the model start year of 2010 for consistency with the future deposition and climate scenarios.

Future Deposition and Climate Scenarios

As detailed in Clark et al. (2023), total Deposition estimates (TDEP) for 2009-2011 served as the source of the current N and S deposition estimates (Schwede and Lear 2014). Future deposition scenarios were from CMAQ v5.0.2 (Zhang et al. 2019) total N and S deposition estimates applied as scaling factors (determined for each plot) to TDEP to estimate reductions in deposition associated with the policy-based reductions in emissions.

Constant deposition scenario (D_0, Figure 1) – this scenario represented the plot-specific 3-year average (2009-2011) N and S deposition levels held constant from 2010-2100.

N deposition reduction scenario (D_N, Figure 1) – This scenario represented policy-based anticipated reductions in N deposition while maintaining S deposition at 2009-2011 levels. Reductions in total N deposition were based on the differences between two years (2011 and 2028) from the CMAQ model developed in Zhang et al. (2019) for total N deposition converted into plot-specific % change in deposition. These represented the best available estimates for anticipated changes in N deposition for the U.S. to support policymaking at the time (EPA 2013b, a). These % changes in deposition were then applied to the plot-specific TDEP N deposition (D_0), as linear declines in annual TDEP N deposition from 2011 to 2028. Total N deposition in 2028 served as the estimate of annual N deposition from 2029 to 2100. Total S deposition remained at the 2009-2011 level for the full simulation (2010-2100).

S deposition reduction scenario (D_S, Figure 1) – Similar to D_N, this scenario represented policy-based reductions in S deposition from 2010-2028 while maintaining N deposition at 2009-2011 levels. S deposition after 2028 was held constant.

N and S deposition reduction scenario (D_{NS}, Figure 1) – This scenario represented policy-based reductions in both N and S deposition per the above procedures from D_N and D_S.

The five climate scenarios included current climate and four future climate scenarios. Each of these provided estimates of mean annual precipitation and average annual temperature that were then used in the growth and survival equations above (eq. 4-8) for the forest model. Climate normals from PRISM (http://www.prism.oregonstate.edu/) served as the source of current temperature and precipitation estimates for each plot. The four future climate scenarios were based on four different Earth System Models (ESMs) from two IPCC AR5 Representative Concentration Pathway (RCP) emission scenarios (Figure 1). At the time of the analysis IPCC AR6 estimates were not yet available. The IPCC AR5 estimates have been statistically downscaled as Localized Constructed Analogs (LOCA) datasets (Pierce et al. 2014) to represent changes of potential temperatures and precipitation at locations across the CONUS. Changes in climate associated with each of these future scenarios were applied as scaling factors

(determined for each plot) to the PRISM data to estimate changes in annual temperature and precipitation predicted by the future scenarios.

Constant climate scenario (CC, Figure 1) – this scenario represented recent climate maintained over time and consisted of plot-specific 30-year average (1981-2010) 4-km PRISM temperature and precipitation estimates repeated from 2010-2100.

Modest climate change scenario ($C_{4.5}$, Figure 1) – This scenario represented potential future climate from RCP 4.5, using a central estimate from the collection of models available (Figure 1). At each plot location, the differences between 10-year averages for 2006–2015 and 2090–2099 of the modeled precipitation and temperature were extracted. These estimates were used to calculate the percent change in precipitation and degree change of temperature projected for at each plot. These changes (percent for precipitation and degrees for temperature) were then be applied to the plot-specific PRISM "constant" climate conditions, as linear changes in temperature and precipitation from 2010 to 2100.

Moderate climate change scenario ($C_{8.5, mod}$, Figure 1) - This scenario represented potential future climate from RCP 8.5, using an estimate from the cooler end of the temperature range and moderate precipitation for RCP 8.5 (Figure 1). A linear change for temperature and precipitation from 2010 to 2100 was then estimated for each plot using the same methods as described above for $C_{4.5}$.

Severe climate change scenario, wet ($C_{8.5, wet}$, Figure 1) - This scenario represented potential future climate from RCP 8.5, using an estimate from the wetter end of the precipitation range (Figure 1). A linear change for temperature and precipitation from 2010 to 2100 was then estimated for each plot using the same methods as described above for $C_{4.5}$.

Severe climate change scenario, dry ($C_{8.5, dry}$, Figure 1) - This scenario represented potential future climate from RCP 8.5, using an estimate from the drier end of the precipitation range (Figure 1). A linear change for temperature and precipitation from 2010 to 2100 was then estimated for each plot using the same methods as described above for $C_{4.5}$.

Total annual N and S deposition, annual precipitation, and average annual temperature for the deposition and climate scenarios were converted into 10-year averages (2010-2019….2080-2089). These 10-year deposition, precipitation, and temperature averages served as the 20 deposition – climate scenario input data for the Forest Composition Model (described further below).

Forest Model

The species-specific growth and survival equations published in (Horn et al. 2018) were applied to the trees in the initial tree database. We used the same modeling approach as in (Van Houtven et al. 2019), but with more species and for the CONUS. Only tree species with $\geq$2000 records (i.e., individual tree data) for the growth and survival models were examined, and only the response curves from the best statistical models were used. Therefore, only 94 of the 352 species included in the initial tree database were modeled, totaling 2.6 million trees. The 94 modeled species were found on 120,159 of the 124,731 FIA plots (i.e., 96.3%) in the initial tree database, and on average, represented 93.2% of plot basal area (Figure S1).

The initial tree database served as the source of the starting tree size and sub-plot basal area model input data, and the deposition and climate scenarios (previously described) were the sources of the precipitation, temperature, and N and S deposition estimates. All model input data were at the plot- or

tree-level. Starting tree size was the aboveground biomass (kg C) of the tree at the beginning of the 10-year time step. Sub-plot basal area was calculated as the sum of the basal area of all modeled trees (that increase in size and diameter with each time step) and non-modeled trees. Non-modeled tree biomass (i.e., those of species other than the 94 modeled species) were held constant at the 2010 biomass values). This approach was also applied to the calculations of sub-plot basal area of all trees larger than the tree of interest, as needed.

The forest model estimated changes in above-ground biomass (i.e., growth) and survival in 10-year time steps from 2010-2100 using the best models from Horn et al. (2018) for each of the 20 scenarios (Figure 1). For each time step, individual tree growth and survival (for the 94 species) were modeled at the sub-plot level, with individual tree biomass and proportion of surviving individuals at the end of the previous time step serving as the starting conditions for the next time step. Growth was modeled as an annual rate (using starting tree size and basal area estimates and 10-year average deposition and climate estimates) multiplied by 10. Survival was modeled as a 10-year probability. Only modeled trees grew and died. The remaining 258 non-modeled tree species are much rarer across the landscape and remained the same size for the full simulation.

In addition, to prevent forecasting growth and survival beyond the empirical records used to establish the models, the growth and survival estimates were restricted to the observed ranges from (Horn et al. 2018)[1]. Once conditions (tree size, N deposition, S deposition, precipitation, temperature, sub-plot basal area and sub-plot basal area of all trees larger than the tree of interest) were outside the training data ranges, annual growth and survival were held constant at the species-specific upper or lower limit for that parameter and that tree was flagged. Thus, we did not extrapolate the response curves beyond the data from which they were derived. Likewise, to prevent individual trees from growing beyond observed sizes, all trees were modeled to stop growing (i.e., individual tree growth of a species was set to 0 kg/yr) once they reached the largest recorded biomass for that species within the USFS FIA database. However, only 0.05-0.11% of the trees (expanded to county level) reached their species-specific maximum biomass and ceased growth during the 2010–2100 period. Many trees, however, reached the edges of the training data for temperature and deposition (discussed below). Sub-plots exceeding recorded basal areas was also a concern, but in 2100 none of the plots had a basal area greater than the largest sub-plot basal area recorded in the FIA database. Lastly, predicted survival rates resulted in some trees being reduced to less than 1 individual at the county level. Similarly, very low trees per hectare counts (approximate 0.00025 trees per ha) for an individual tree resulted in some survival equation predictions returning an "error". In these situations, the tree was recorded as dead, thereby representing 0 trees per hectare (tph) at the county level in the next model time step.

Estimating changes in forest composition

Abundances by species were expanded to the county using standard FIA expansion factors (USFS 2018) and then summed to the CONUS. Differences in composition between scenarios were assessed for each county comparing relative abundances for each species in a county in the final decade of the simulation (i.e., 2090-2100). The compositional difference (CD) for one county at end-of-century between two scenarios was estimated as the pairwise sum of minimum percent abundances by species (equation S1).

$$CD_{j,k,l}\ (\%) = 100 - \sum_i^N \min\left(Ps_{i,j,k}, Ps_{i,j,l}\right) \quad \text{(eq. S1)}$$

[1] The empirical data ranges were from the growth equations because they are the most conservative.

Where $CD_{j,k,l}$ is the compositional difference in county j between scenarios k and l, N is the number of species in county j, and $Ps_{i,j,k}$ and $Ps_{i,j,l}$, are the relative abundances (in percent) of species i and county j, comparing scenarios k and l. For example, if a county had three species under one scenario (e.g., Ps_1=50%, Ps_2=25%, Ps_3=25%) and three under another scenario that only partly overlapped (e.g., Ps_1=80%, Ps_2=10%, Ps_4=10%), the compositional similarity would be 60% (50% from Ps_1, 10% from Ps_2, and none from Ps_3 or Ps_4 which aren't shared between scenarios for that county), and the compositional difference would be 40%.

Driver	Symbol	Description	
**Deposition**	D_0	Total N and S deposition from TDEP averaged for 2009-2011 for plot-specific locations and held constant from 2010-2100.	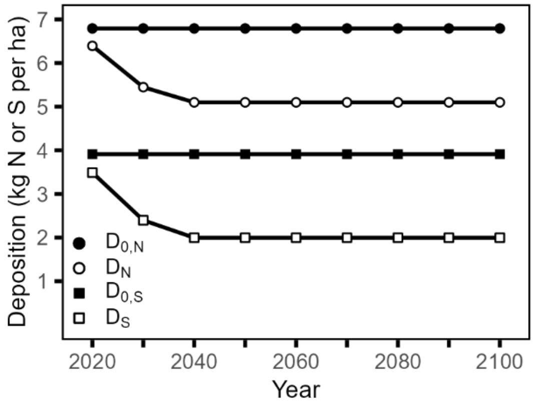
	D_N	Anticipated reductions in N deposition to 2025 based on CMAQ 5.0.2 with constant S deposition	
	D_S	Anticipated reductions in S deposition to 2025 based on CMAQ 5.0.2 with constant N deposition	
	D_{NS}	Anticipated reductions in both N and S deposition to 2025 based on CMAQ 5.0.2.	
**Climate**	CC	Climate held constant from 2010-2100 at location-specific 30-year normal using PRISM	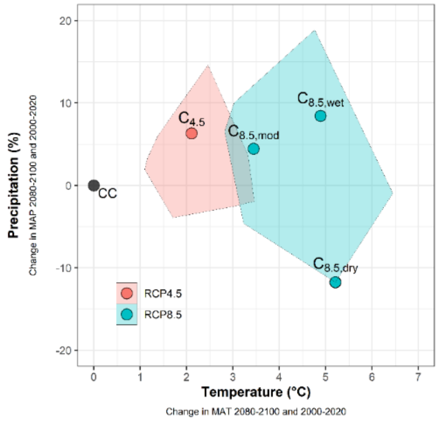
	$C_{4.5}$	Modest climate change using IPCC AR5 RCP 4.5 scenario and model CNRM-CM5.	
	$C_{8.5,mod}$	More climate change (wet and warm) using IPCC AR5 RCP 8.5 and model GFDL-ESM2G	
	$C_{8.5,wet}$	More climate change (wet and very hot) using IPCC AR5 RCP 8.5 and model CESM-CAM5	
	$C_{8.5,dry}$	More climate change (dry and very hot) using IPCC AR5 RCP 8.5 and model IPSL-CMA-MR	

Figure S1: The deposition and climate scenarios used in the study. The table (left) describes the scenarios examined (all 20 combinations of climate and deposition scenarios were run). Throughout, combined scenarios are listed together where appropriate (e.g., for current deposition and climate as D_0/CC). The deposition linear plot (top right) shows the scenarios of average CONUS deposition through time. The climate biplot (bottom right) summarizes the average CONUS changes between 2000-2020 and 2080-2100 for the scenarios explored here (points). For reference also shown are the range from the full ensemble of IPCC AR5 models for RCP 4.5 (red polygon) and 8.5 (blue polygon).

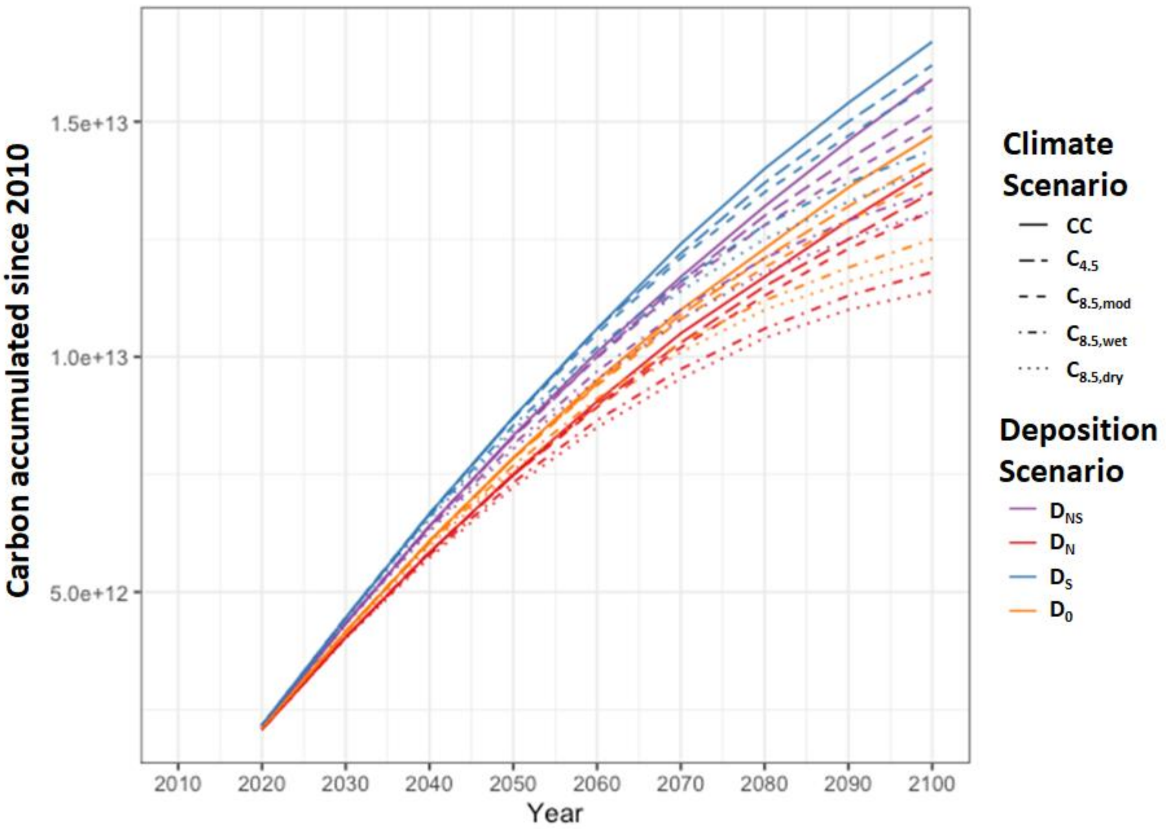

Figure S2 Projected new carbon accumulation in aboveground biomass of existing cohort of trees through 2100, summed across the contiguous United States. Line type indicates climate scenarios and colors indicate deposition scenarios.

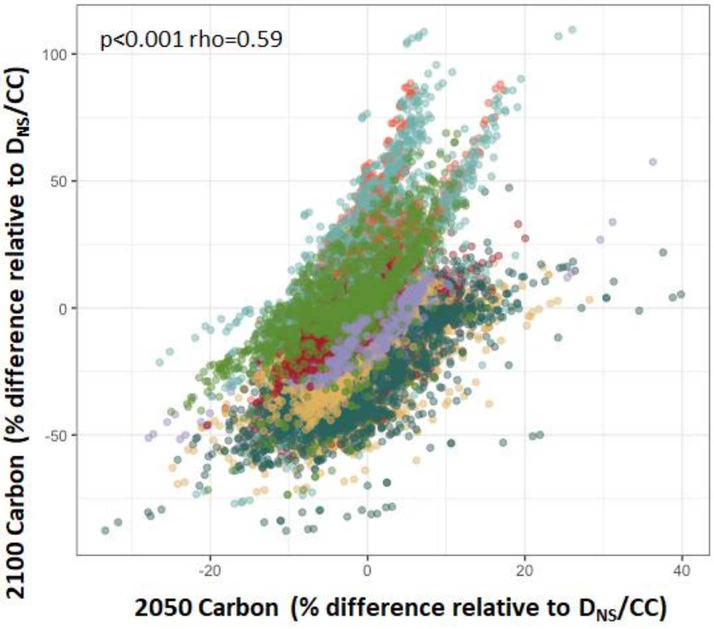

Figure S3: Projected county-level aboveground forest carbon response in 2100 as a function of projected county-level aboveground forest carbon response in 2050. Response values are relative to the reference scenario (D_{NS}/CC). Spearman correlation results are inset. Colors indicate USGCRP National Climate Assessment region as in Figure 3.

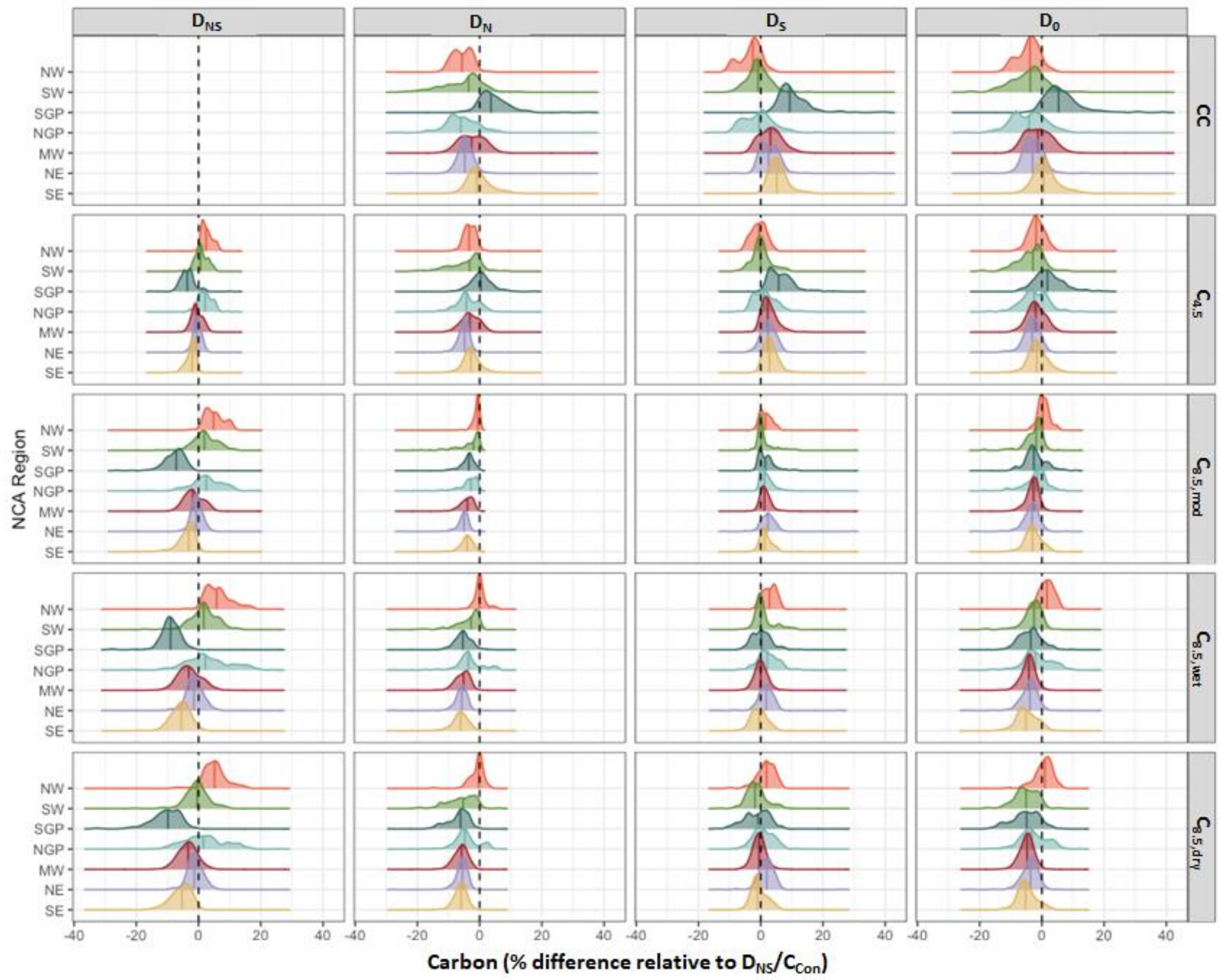

Figure S4: Density plots of projected county-level aboveground forest carbon responses in 2050 across all model scenarios. Response values are relative to the reference scenario (D_{NS}/CC). Y axis and colors indicate USGCRP National Climate Assessment region. Solid vertical lines indicate regional median.

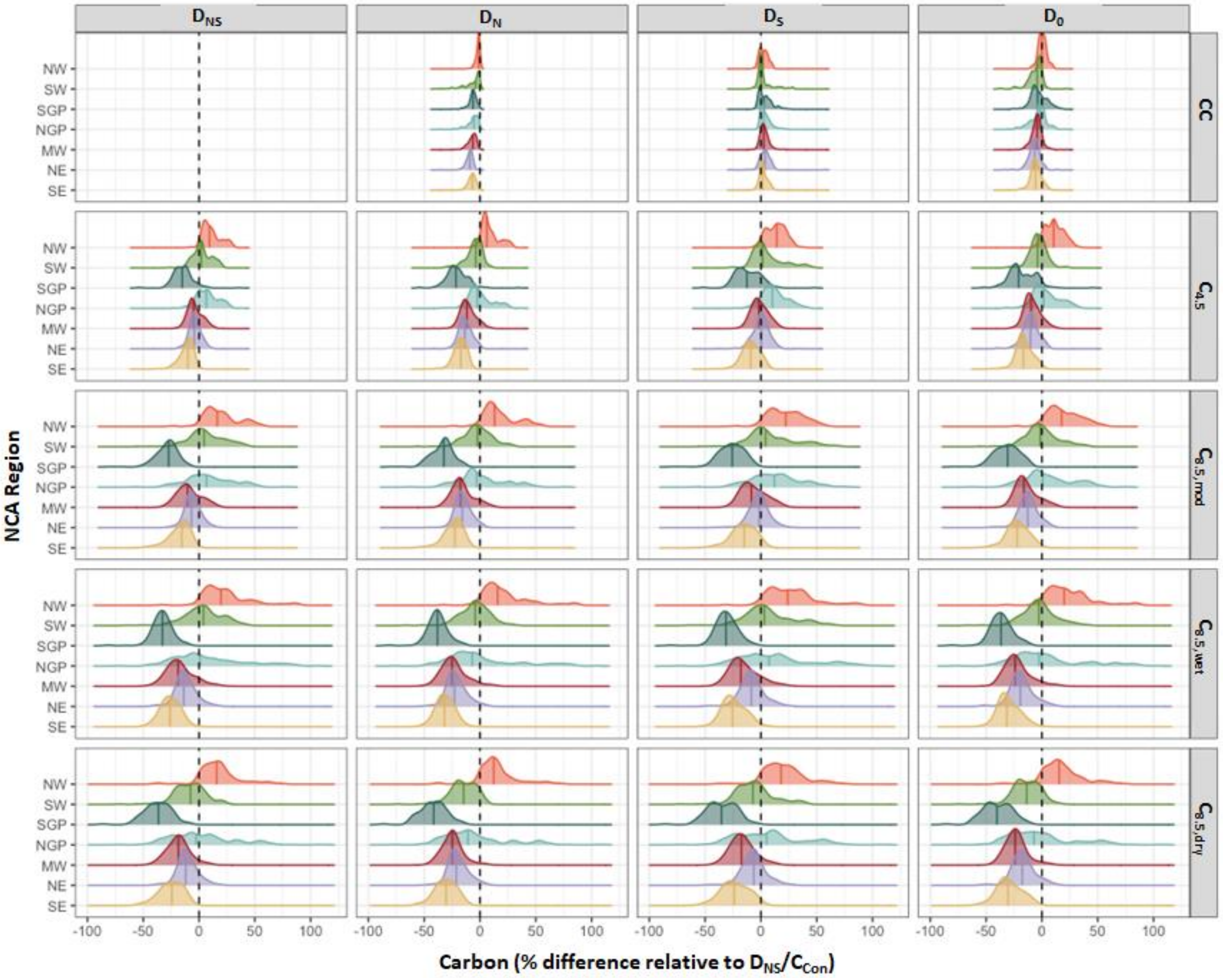

Figure S5: Density plots of projected county-level aboveground forest carbon responses in 2100 across all model scenarios. Response values are relative to the reference scenario (D_{NS}/CC). Y axis and colors indicate USGCRP National Climate Assessment region. Solid vertical lines indicate regional median.

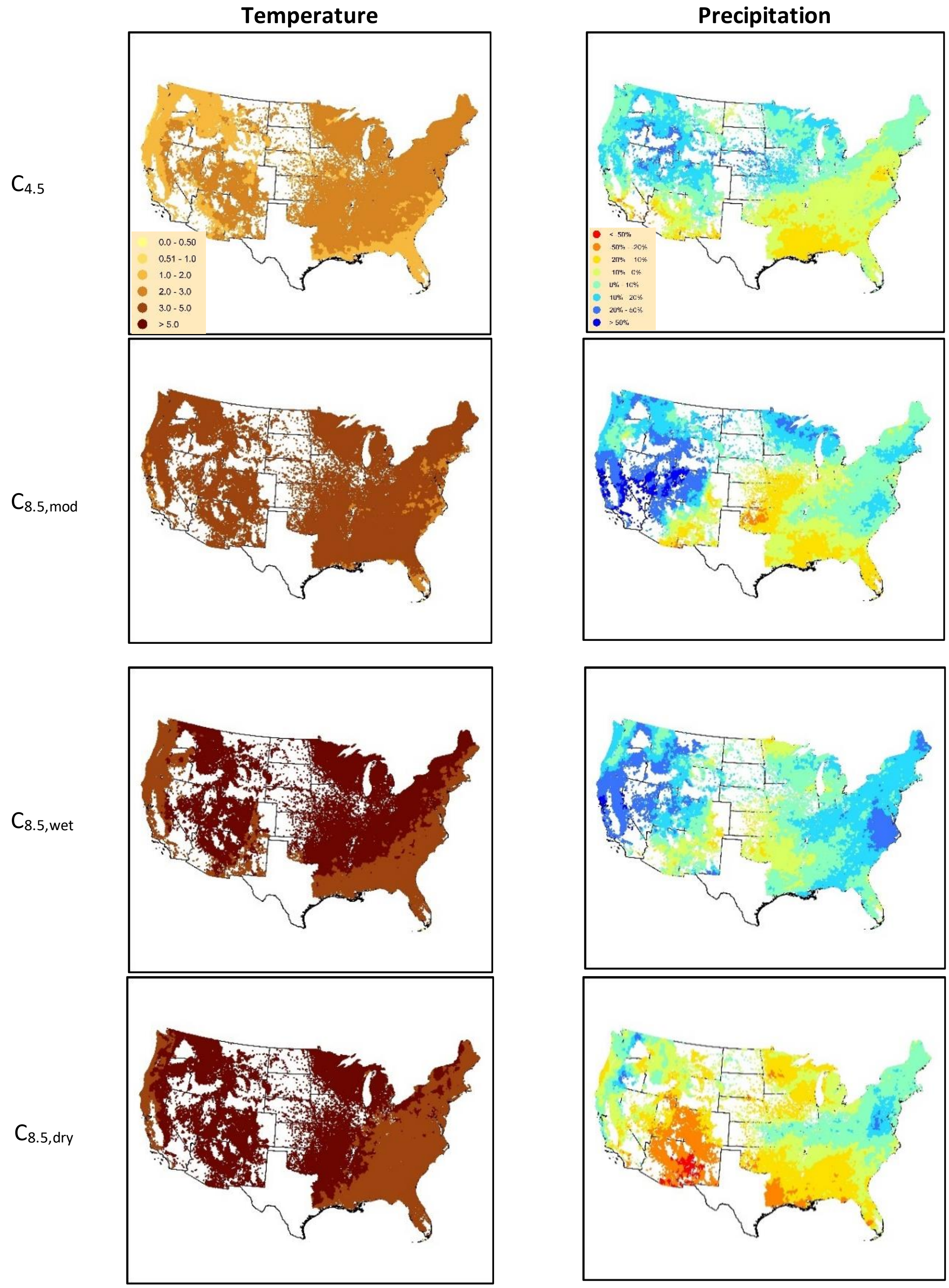

Figure S6: Changes in temperature and precipitation in 2100 relative to the reference scenario (CC). Temperature is shown as a difference (scenario – CC) and precipitation is shown as a percent difference (100*(scenario – CC)/CC)). Legends for temp and precip are in the top row.

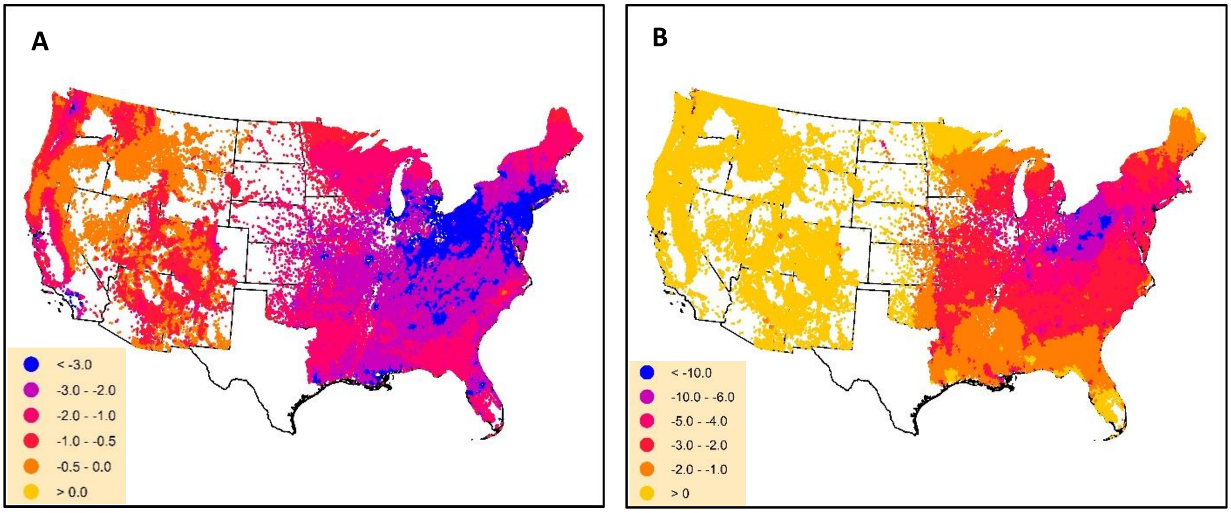

Figure S7: Reductions in N deposition (A: D_N-D_0; kg N ha^{-1} yr^{-1},) and S deposition (B: D_S-D_0; kg S ha^{-1} yr^{-1}) in 2100 relative to the baseline reference D_0.

A

Variable	Estimate	p value	Effect size (d)
Temperature	3.182	<0.001	0.80
Precipitation	3.208	<0.001	0.80
Temp*Precip	-0.316	<0.001	-0.08
NW	15.41	0.008	3.86
SW	31.91	<0.001	7.99
SGP	62.21	<0.001	15.57
NGP	13.48	0.022	3.37
MW	-14.23	0.017	-3.56
SE	33	<0.001	8.26
NW*Temp	-0.2951	.576	-0.07
SW*Temp	-3.424	<0.001	-0.86
SGP*Temp	-6.919	<0.001	-1.73
NGP*Temp	-1.861	0.001	-0.47
MW*Temp	-0.4692	0.375	-0.12
SE*Temp	-3.901	<0.001	-0.98
NW*Precip	-2.785	<0.001	-0.70
SW*Precip	-2.749	<0.001	-0.69
SGP*Precip	-0.7726	0.252	-0.19
NGP*Precip	-0.1999	0.719	-0.05
MW*Precip	3.45	<0.001	0.86
SE*Precip	-0.5805	0.293	-0.15
NW*Temp*Precip	0.2403	<0.001	0.06
SW*Temp*Precip	0.3328	<0.001	0.08
SGP*Temp*Precip	0.3361	<0.001	0.08
NGP*Temp*Precip	0.1621	0.002	0.04
MW*Temp*Precip	-0.1277	0.006	-0.03
SE*Temp*Precip	0.2039	<0.001	0.05

B

Variable	Estimate	p value	Effect size (d)
Temperature	4.272	<0.001	0.37
Precipitation	6.072	<0.001	0.53
Temp*Precip	-0.5325	<0.001	-0.05
NW	14.33	0.196	1.25
SW	52.01	<0.001	4.54
SGP	85.36	<0.001	7.45
NGP	14.71	0.1875	1.28
MW	-27.25	0.016	-2.38
SE	91.95	<0.001	8.03
NW*Temp	1.034	0.201	0.09
SW*Temp	-5.33	<0.001	-0.47
SGP*Temp	-9.187	<0.001	-0.80
NGP*Temp	-4.09	<0.001	-0.36
MW*Temp	-1.347	0.097	-0.12
SE*Temp	-7.74	<0.001	-0.68
NW*Precip	-4.489	<0.001	-0.39
SW*Precip	-4.478	<0.001	-0.39
SGP*Precip	-0.6432	0.606	-0.06
NGP*Precip	0.2719	0.798	0.02
MW*Precip	6.011	<0.001	0.52
SE*Precip	-3.012	0.004	-0.26
NW*Temp*Precip	0.3692	<0.001	0.03
SW*Temp*Precip	0.5737	<0.001	0.05
SGP*Temp*Precip	0.452	<0.001	0.04
NGP*Temp*Precip	0.5805	<0.001	0.05
MW*Temp*Precip	-0.1244	0.075	-0.01
SE*Temp*Precip	0.4174	<0.001	0.04

Table S1: Results of ANOVAs of linear mixed effects models for projected aboveground forest carbon response as a function of mean temperature, mean precipitation, NCA region and their interactions, as fixed effects, and county, as a random effect. Model estimate, p value, and estimated effect size (d) for each fixed effect term are recorded for the 2050 model (A) and the 2100 model (B). Effect size cells are colored with yellow indicating small effects ($0.4>|d|\geq0.2$), orange indicating medium effects ($0.8>|d|\geq0.4$), and red indicating large effects ($|d|\geq0.8$) for variables which have p values less than 0.001.
